# Laboratory colonization by *Dirofilaria immitis* alters the microbiome of female *Aedes aegypti* mosquitoes

**DOI:** 10.1186/s13071-020-04218-8

**Published:** 2020-07-13

**Authors:** Abdulsalam Adegoke, Erik Neff, Amie Geary, Montana Ciara Husser, Kevin Wilson, Shawn Michael Norris, Guha Dharmarajan, Shahid Karim

**Affiliations:** 1 Center for Molecular and Cellular Biosciences, School of Biological, Environmental, and Earth Sciences, The University of Southern Mississippi, Hattiesburg, MS 39406 USA; 2 Savannah River Ecology Laboratory, University of Georgia, P.O. Drawer E, Aiken, SC 29802 USA; 3 Warnell School of Forestry and Natural Resources, University of Georgia, Athens, GA 30602 USA

**Keywords:** Mosquitoes, *Aedes aegypti*, Microbiome, Metagenome, *Dirofilaria immitis*, Dog heartworm

## Abstract

**Background:**

The ability of blood-feeding arthropods to successfully acquire and transmit pathogens of medical and veterinary importance has been shown to be interfered with, or enhanced by, the arthropod’s native microbiome. Mosquitoes transmit viruses, protozoan and filarial nematodes, the majority of which contribute to the 17% of infectious disease cases worldwide. *Dirofilaria immitis*, a mosquito-transmitted filarial nematodes of dogs and cats, is vectored by several mosquito species including *Aedes aegypti*.

**Methods:**

In this study, we investigated the impact of *D. immitis* colonization on the microbiome of laboratory reared female *Ae. aegypti*. Metagenomic analysis of the V3–V4 variable region of the microbial *16S* RNA gene was used for identification of the microbial differences down to species level.

**Results:**

We generated a total of 1068 OTUs representing 16 phyla, 181 genera and 271 bacterial species. Overall, in order of abundance, Proteobacteria, Bacteroidetes, Actinobacteria and Firmicutes were the most represented phylum with *D. immitis-*infected mosquitoes having more of Proteobacteria (71%) than uninfected mosquitoes (56.9%). An interesting finding in this study is the detection of *Klebsiella oxytoca* in relatively similar abundance in infected and uninfected mosquitoes, suggesting a possible endosymbiotic relationship, and has been previously shown to indirectly compete for nutrients with fungi on domestic housefly eggs and larvae. While *D. immitis* colonization has no effect on the overall species richness, we identified significant differences in the composition of selected bacterial genera and phyla between the two groups. We also reported distinct compositional and phylogenetic differences in the individual bacterial species when commonly identified bacteria were compared.

**Conclusions:**

To the best of our knowledge, this is the first study to understand the impact of a filarial infection on the microbiome of its mosquito vector. Further studies are required to identify bacteria species that could play an important role in the mosquito biology. While the microbiome composition of *Ae. aegypti* mosquito have been previously reported, our study shows that in an effort to establish itself, a filarial nematode modifies and alters the overall microbial diversity within its mosquito host.
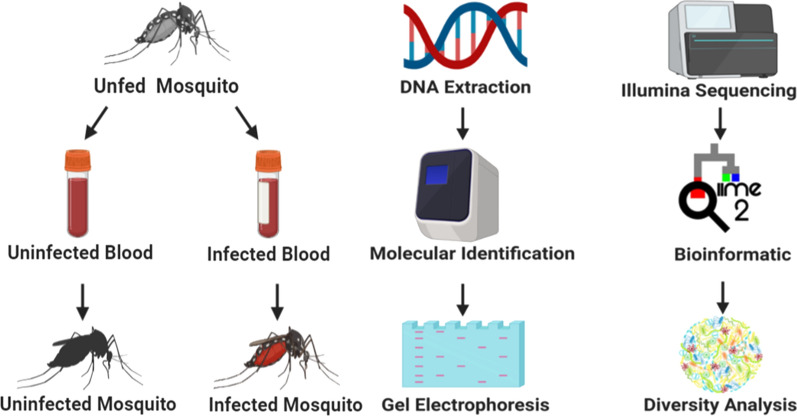

## Background

*Dirofilaria immitis*, responsible for canine respiratory and associated heartworm disease, is a filarial nematode that mainly infects wild and domestic canids, such as dogs and coyotes [1]. It causes serious life-threatening disease of canines and other domestic pets in which adult worms lodge in the cardiopulmonary circulation causing cardiac necrosis which leads to ischemic hypoxia and a total shutdown of the respiratory and cardiovascular system [2]. While primarily a parasite of veterinary importance, human infection has also been reported, albeit rarely [3]. With over 100,000 dogs affected annually, *D. immitis* has been identified as the most important parasite affecting dogs in the USA [4]. Adult female *D. immitis* parasites release microfilariae (mf) into the blood stream of the vertebrate host, usually a dog, which are subsequently taken up by adult female mosquitoes during their blood meal [1, 5]. Microfilariae in the blood enter the midgut of the mosquito during feeding and subsequently migrate to the Malpighian tubules. In the Malpighian tubules of the mosquito mf develop into first-stage larvae and molt twice to reach their infective third-stage (L3), which migrate to the head and proboscis of the mosquito and can infect the next dog during the next blood meal [1, 5]. The time it takes for the parasite to develop, known as the extrinsic incubation period (EIP), averages 14 days within this mosquito [6].

Beyond the USA, canine dirofilariasis also poses significant problems in dogs in Australia, Europe and Asia [7]. The epidemiology and maintenance of *D. immitis* have been made significant by the abundance of the mosquito vectors with over 70 species of mosquitoes, having been reported to transmit *D. immitis* [8]; of these, 28 are found in the USA [9]. The southeastern USA have been shown to have an increased incidence of canine heartworm disease when compared to other states [10], as about half of the mosquito species known to transmit *D. immitis* are found in the southeastern USA [11], one of which is the mosquito *Aedes aegypti* [12].

In addition to being an important vector of filarial nematodes such as *D. immitis*, mosquitoes and other arthropod vectors also host highly complex and diverse microbial communities. Members of these microbial communities have obligate relationships with mosquitoes and are maintained vertically through mosquito generations [13] or acquired from the environment [14, 15]. The microbiome of mosquitoes has been shown to help maintain normal midgut functions, as well as interfering or aiding vector competence of the mosquito [16, 17]. As previously shown, pathogen susceptibility and vector competence of an arthropod can be modulated by the microbial composition within the arthropod vector [18]. *Aedes aegypti* is the competent vector of many pathogens of humans (Zika, dengue, chikungunya viruses [19–21] and animals (e.g. *D. immitis*; [22]).

Previous studies have demonstrated significant success in reducing vector competence of mosquitoes for pathogenic microbes by taking advantage of the mosquito microbiome and pathogen interactions [23, 24]. It is a fact that pathogens must compete with the native microbial community in their vector host to propagate and facilitate subsequent transmission to a susceptible host [18, 25]. Hence, understanding the pathogen-microbiome interaction in vectors of significant public health importance is important.

The prevention and control of canine heartworm disease have primarily focused on the use of therapeutics such as ivermectin and other macrocyclic lactones. While these therapeutics have proven to be effective in killing and preventing the establishment of the third- and early fourth-stage larva [26], recent studies have shown significant resistance against these drugs of choice [27, 28]. Understanding the role(s) played by the mosquito microbiome in facilitating or interfering with *D. immitis* colonization and subsequent transmission will add to existing knowledge on the control of mosquito-transmitted filarial nematodes.

The present study compared the microbiome changes in the mosquito *Ae. aegypti*, infected with *D. immitis* under laboratory conditions. This study provides an exciting opportunity to advance our knowledge of the major changes in the microbiome of *Ae. aegypti* following the acquisition of *D. immitis*. This research seeks to address the question “Does *D. immitis* infection of *Ae. aegypti* mosquito alters its overall microbial richness and abundance?”

## Methods

### Materials

Unless stated otherwise, equipment and chemicals used for this study were either products of Thermo Fisher Scientific (Grand Island, NY, USA) or Bio-Rad (Hercules, CA, USA).

### Mosquito rearing and maintenance

The mosquito used for investigation was the *Ae. aegypti* Liverpool Blackeye strain, a highly susceptible mosquito strain to *D. immitis* used predominately in research for *Aedes* spp. [29]. Although *Ae. aegypti* is just one of 28 mosquito species reported to vector *D. immitis* in the USA, the relative expertise on this particular species limited us to its use as our study organism. *Aedes aegypti*, originally obtained from the Filariasis Research Reagent Resource Center (FR3) [30], were raised under standard laboratory conditions: temperature of 27 °C, relative humidity of 80 ± 5%, and a 12:12-hour light:dark diurnal cycle [31].

### Mosquito infection with *D. immitis*

Adult female mosquitoes, five days post-emergence were blood-fed using an artificial membrane feeder. One day prior to membrane-feeding 31 female mosquitoes were transferred to ~500 ml plastic containers with mesh tops (henceforth “cages”). Females were starved of sugar for 12 h and deprived of water for 4 h prior to blood-feeding. Mosquitoes in each cage (31 each) were allowed to feed for 2 h or until repletion on a Parafilm membrane stretched over an inverted water-jacketed glass membrane feeder maintained at 40 °C. Each feeder was filled with 200 µl of dog blood infected or uninfected with *D. immitis*. The level of parasitaemia in the infected blood was estimated to be 4500 mf/ml as previously reported [31]. The dog blood was obtained from FR3.

### PCR-based confirmation of *Dirofilaria immitis* detection

Prior to screening for *D. immitis* infection from mosquitoes, DNA from individual mosquito samples was extracted using a DNeasy Blood & Tissue Kit (Qiagen, Germantown, Maryland, USA) and quality was confirmed using a nanodrop machine (Nanodrop One, Thermo Fisher Scientific). All blood-fed (infected and uninfected blood) mosquitoes were screened for *D. immitis* infection irrespective of whether they were fed on infected or uninfected blood by amplifying the *cox*1 gene (656 bp) of the *D. immitis* mitochondrial DNA [32]. Briefly, a 25 µl reaction was set up comprising 1 µl each of the COI-Forward (5′-TGA TTG GTG GTT TTG GTA A-3′) and COI-Reverse (5′-ATA AGT ACG AGT ATC AAT ATC-3′) primers, 12.5 µl of 2× mastermix (New England Biolabs, Ipswich, Massachusetts, USA), 2.5 µl DNA template and 8.5 µl of nuclease-free water. For each cycle that was run, a *D. immitis* infected blood sample and nuclease-free water were simultaneously included as positive and negative controls respectively. The PCR cycle comprised of an initial denaturation step at 94 °C for 5 min and 40 cycles of 94 °C for 1 min, annealing at 50 °C for 2 min and extension at 72 °C for 3 min, followed by a final extension step at 72 °C for 5 min and an infinite hold at 4 °C.

Confirmation of amplification was done by loading the PCR products in a SYBR safe stained gel. Briefly, 2% gel was made by autoclaving a solution of 1× TAE buffer and molecular grade agar. SYBR safe stain (1 µl SYBR safe: 10 ml TAE buffer) was added to the agar solution, poured into a precast gel tray and allowed to cool. To load the samples onto the gel, 6 µl of PCR product was mixed with 4 µl of 6× dye and pipetted into the wells. Lastly, 5 µl of a low molecular weight DNA ladder was loaded onto the gel and the gel could run for 45 min at 100 V. Amplified PCR products were viewed using a Chemidoc gel imager (Additional file 1: Figure S1).

## *16S* rRNA library preparation and sequencing

Six individual mosquito genomic DNA extracts were pooled to make one biological replicate and five biological replicates each of *D. immitis* infected and uninfected pools were prepared for metagenomic analysis. The hypervariable V1-V3 region of the *16S* rRNA gene was PCR amplified using the forward primer 27F (5′-AGR GTT TGA TCM TGG CTC AG-3′) and the reverse primer 519R (5′-GTN TTA CNG CGG CKG CTG-3′) as outlined by the *16S* Illumina’s MiSeq protocol (https://www.mrdnalab.com, Shallowater, TX, USA). Briefly, PCR was performed using the HotStarTaq Plus Master Mix Kit (Qiagen) under the following conditions: 94 °C for 3 min, followed by 30–35 cycles of 94 °C for 30 s, 53 °C for 40 s and 72 °C for 1 min, after which a final elongation step at 72 °C for 5 min was performed. After amplification, PCR products were electrophoresed in 2% agarose gel to determine the success of amplification and the relative intensity of bands. Multiple samples were pooled together in equal proportions based on their molecular weight and DNA concentrations. Pooled samples were purified using calibrated Ampure XP beads. Then the pooled and purified PCR product was used to prepare Illumina DNA library. Sequencing was performed at MR DNA (https://www.mrdnalab.com, Shallowater, TX, USA) on a MiSeq following the manufacturer’s guidelines.

### Sequence analysis

Sequence analysis was carried out using the Quantitative Insights into Microbial Ecology (QIIME 2) pipeline, unless stated otherwise. Briefly, processing of raw fastq files were demultiplexed. The Atacama soil microbiome pipeline was incorporated for quality control of demultiplexed paired-end reads (Additional file 1: Figure S2) using the DADA2 plugin as previously described [33].

Sequence alignment and subsequent construction of phylogenetic tree from representative sequences was performed using the MAFFT v7 and FasTree v2.1 plugin [34] Operational taxonomic assignment was performed using the qiime2 feature-classifier plugin v7.0 which was previously trained against the SILVA 132 database preclustered at 99%. Tables representing operational taxonomic units (OTUs) and representative taxonomy were exported from R and used for diversity metric analysis using the Microbiome Analyst web-based interface [35, 36]. Raw data from this analysis were deposited and assigned the GenBank BioProject number #PRJNA606536.

### Alpha diversity

To establish whether alpha diversity differs across mosquito samples, reads were transformed and low abundance OTUs were filtered from the datasets. The Observed OTU metric was used to estimate species richness by identifying unique OTUs present across the mosquito groups, while the Shannon index was used to estimate both richness and evenness.

### Beta diversity

To compare the differences in the microbiome between mosquito groups based on measures of distance or dissimilarity, dissimilarity matrix was generated from log-transformed sequence data and ordination of the plots were visualized using both the principal coordinates analysis (PCoA) and the non-metric multidimensional scaling (NMDS). The matrix used in calculating beta diversity includes the Bray-Curtis and unweighted UniFrac distance matrix.

### Statistical analysis

To test if species richness and diversity was significant, the Mann-Whitney or Kruskal-Wallis tests was applied to both alpha diversity and classical univariate statistical comparisons analysis, while the significance of beta diversity analysis was determined using the permutational MANOVA (PERMANOVA) test [35, 36].

## Results

### *Dirofilaria immitis* effectively colonizes *Aedes aegypti* mosquitoes under laboratory conditions

A total of 31 mosquitoes were artificially infected to generate *D. immitis-*infected mosquito. PCR analysis and confirmation of infection status using gel electrophoresis revealed an infection prevalence of 87% (Additional file 1: Figure S1).

### Microbiome composition

Analysis of the demultiplexed paired-end-reads generated a total of 602,502 reads which ranged from 31,861 to 110,235, with an average of 54,758 reads per mosquito. Mosquitoes infected with *D. immitis* had the highest number of reads (382,714) compared to uninfected mosquitoes with a total of 219,788 reads. Taxonomic classification using the SILVA 132 reference database (99% OTUs full-length sequences) identified 268 operational taxonomic units (OTUs), 11 phyla, 16 genera, and 136 species (Additional file 1: Figure S3).

### *Dirofilaria immitis* infection alters relative abundance of bacteria taxa in *Aedes aegypti*

Taxonomic assignment was performed against the SILVA database to observe for the difference in microbiome composition and relative abundance of bacteria species. Both infected and uninfected mosquitoes possessed similar composition of bacteria taxa with differences observed in the relative abundances of specific bacteria species.

**Fig. 1:**
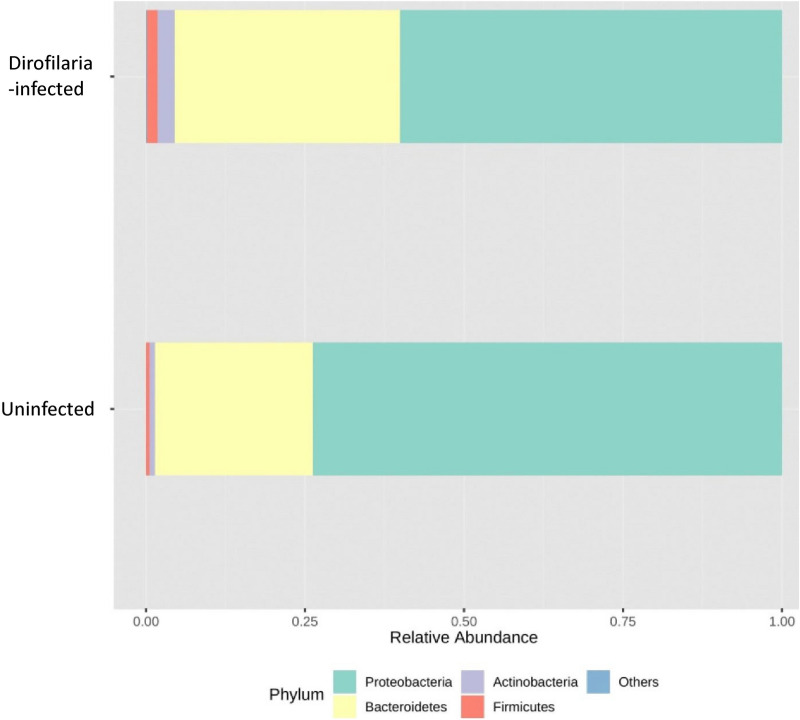
Phylum level distribution. Relative abundance of bacteria at the phylum level as identified in *Dirofilaria immitis-*infected and uninfected *Aedes aegypti* mosquitoes. The phyla Proteobacteria and Bacteroidetes were the most abundant in both groups. Phyla with less than 1% abundance were grouped as “Others”

**Fig. 2:**
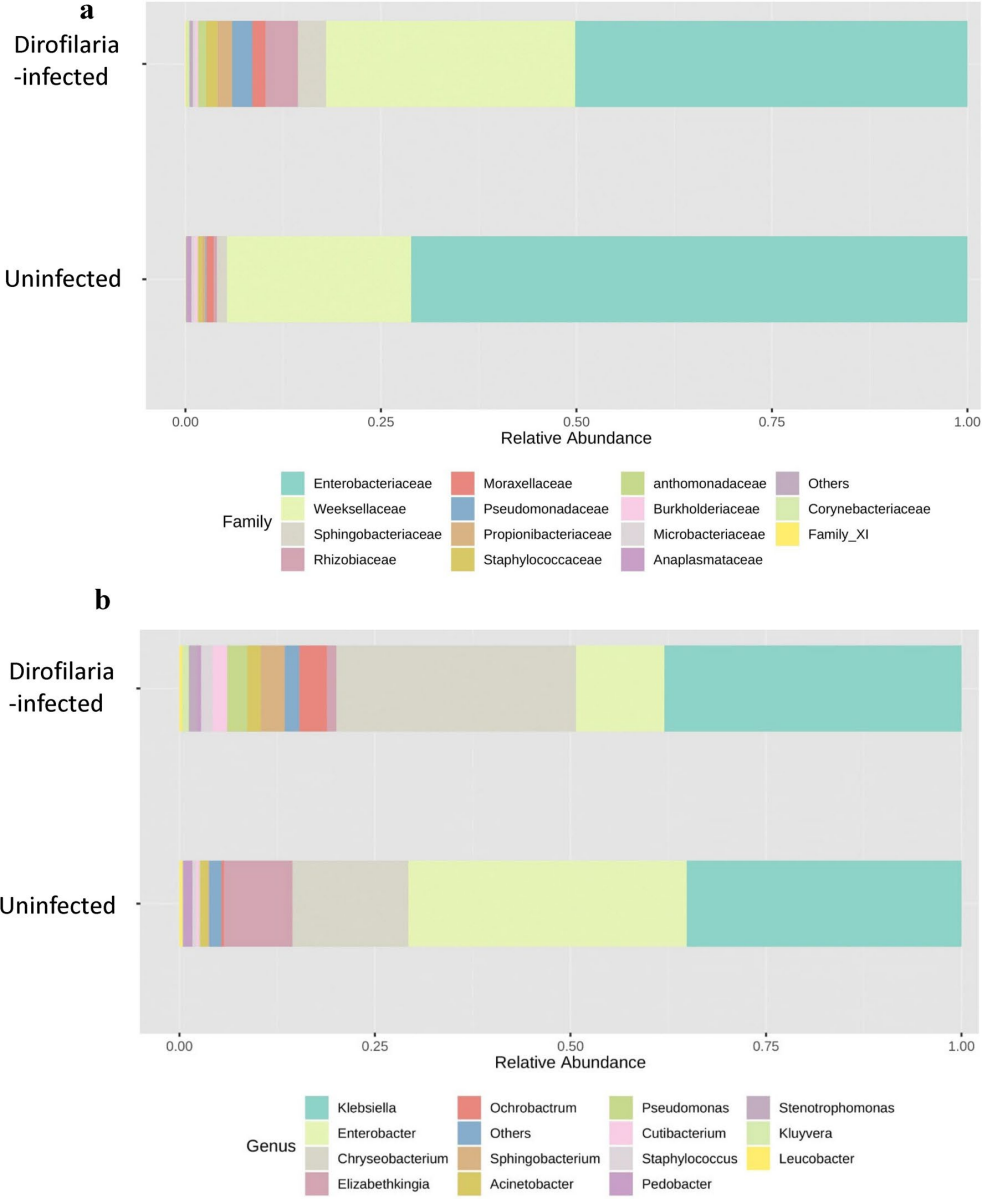
Genus and species level distribution. Relative abundance of bacteria at genus (**a**) and species (**b**) level *Dirofilaria immitis-*infected and uninfected *Aedes aegypti* mosquitoes. Bacteria taxa with less than 1% abundance were grouped as “Others”

The phylum Proteobacteria and Bacteroidetes were present in higher abundance in both mosquito groups. Mosquitoes infected with *D. immitis* had relatively higher abundance of Proteobacteria (71%) with lower amount of Bacteroidetes (27%), while uninfected mosquitoes had lower amount of Proteobacteria (56.9%) and a higher abundance of Bacteroidetes (37%) (Fig. 1). Among the detected bacteria genera, the genus *Klebsiella* were detected at relatively similar abundances in both infected (36.3%) and uninfected (34.6%) mosquitoes (Fig. 2a).

The genera *Ochrobactrum*, *Sphingobacterium* and *Pseudomonas* were present exclusively in uninfected mosquitoes albeit at an abundance of 4.4%, 3.6% and 3%, respectively (Fig. 2). The genera *Enterobacter* (26.1%) and *Elizabethkingia* (9.4%) were in higher abundance in *D. immitis-*infected mosquitoes (Fig. 2a). *Enterobacter hormaechei* (24.4%) and *Elizabethkingia meningoseptica* (9.4%) were more abundant in infected mosquitoes, while *Chryseobacterium indologenes* (27.4%) and *Grimontella senegalensis* (3.9%), were more abundant in uninfected mosquitoes (Fig. 2b). Additional information on the relative bacteria distribution in individual mosquito can be found in Additional file 1: Figures S4–S7.

### *Dirofilaria immitis* infection affects species diversity in *Aedes aegypti*

**Fig. 3:**
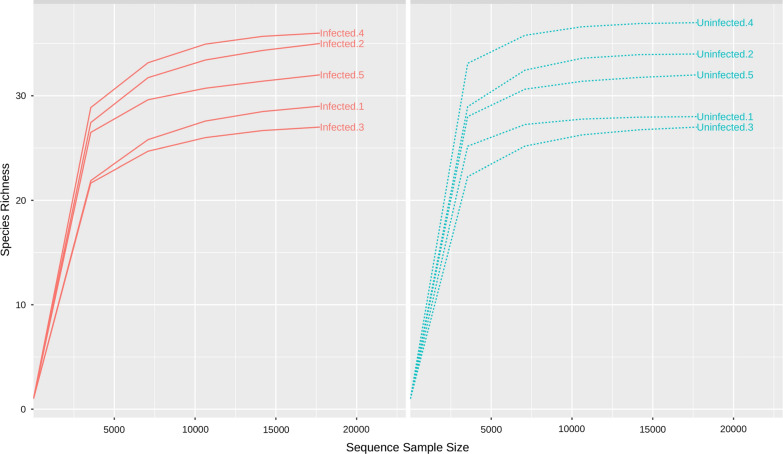
Rarefaction curves. Rarefaction analysis of biological replicates rarefied at a sequenced depth of 20,000. Curves were allowed to reach a plateau so as to prevent trade-off of rarely represented bacterial OTUs. *Dirofilaria immitis-*infected mosquitoes are represented by the red curves, while the blue curves represent the uninfected mosquitoes

Following demultiplexing of paired-end reads and quality control by removing chimeric sequences, we normalized OTU counts for individual biological replicates and a rarefaction curve was generated at a depth of 20,000 (Fig. 3). Adequate depth coverage was reached as evidenced by the individual curves plateauing out on the rarefaction curve.

**Fig. 4:**
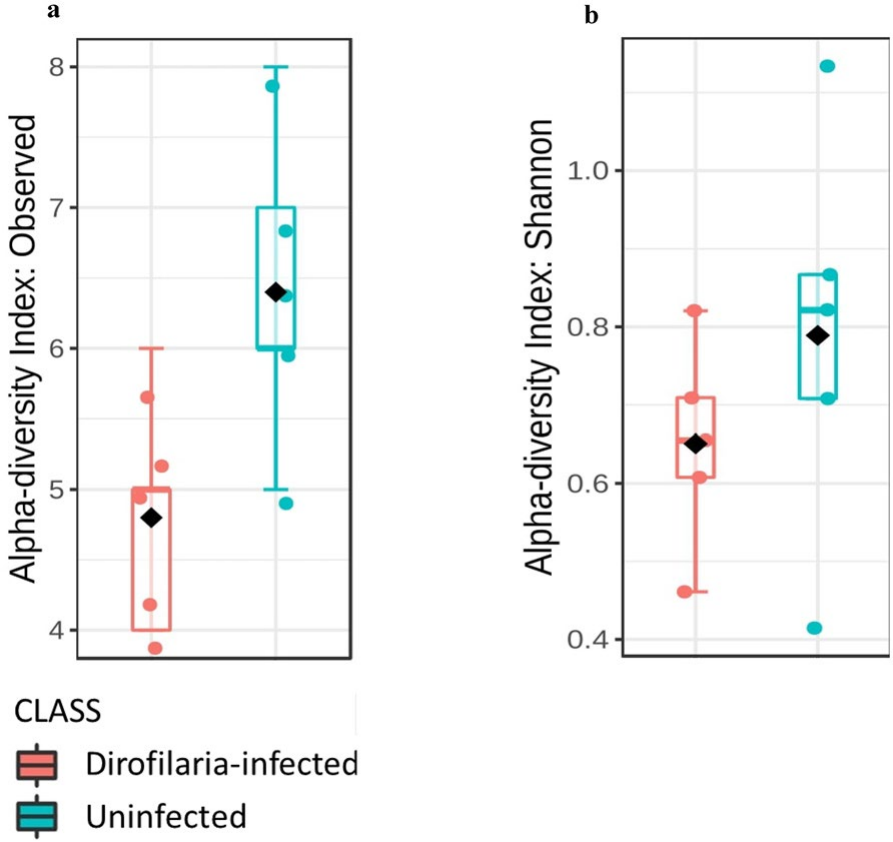
Alpha diversity analysis. Estimation of species richness and evenness using the number of observed OTUs (**a**) (Kruskal–Wallis H-test, *H* = 18.8, *df* = 1, *P* = 0.053) and Shannon diversity index (**b**) (Kruskal–Wallis H-test, *H* = 3.2, *df* = 1, *P* = 0.309). For both measures, mosquitoes infected with *Dirofilaria immitis* exhibited reduced alpha diversity

Our results indicated that phylogenetic diversity, estimated using the Shannon index and number of observed OTUs, was reduced in infected mosquitoes compared against uninfected mosquitoes (Fig. 4a, b). Surprisingly, both metrics of alpha diversity used showed no significance when the Kruskal-Wallis test was applied (Observed OTUs: Mann-Whitney *U* = 3, *P* = 0.053; Shannon index: Mann-Whitney *U* = 7, *P* = 0.309).

**Fig. 5:**
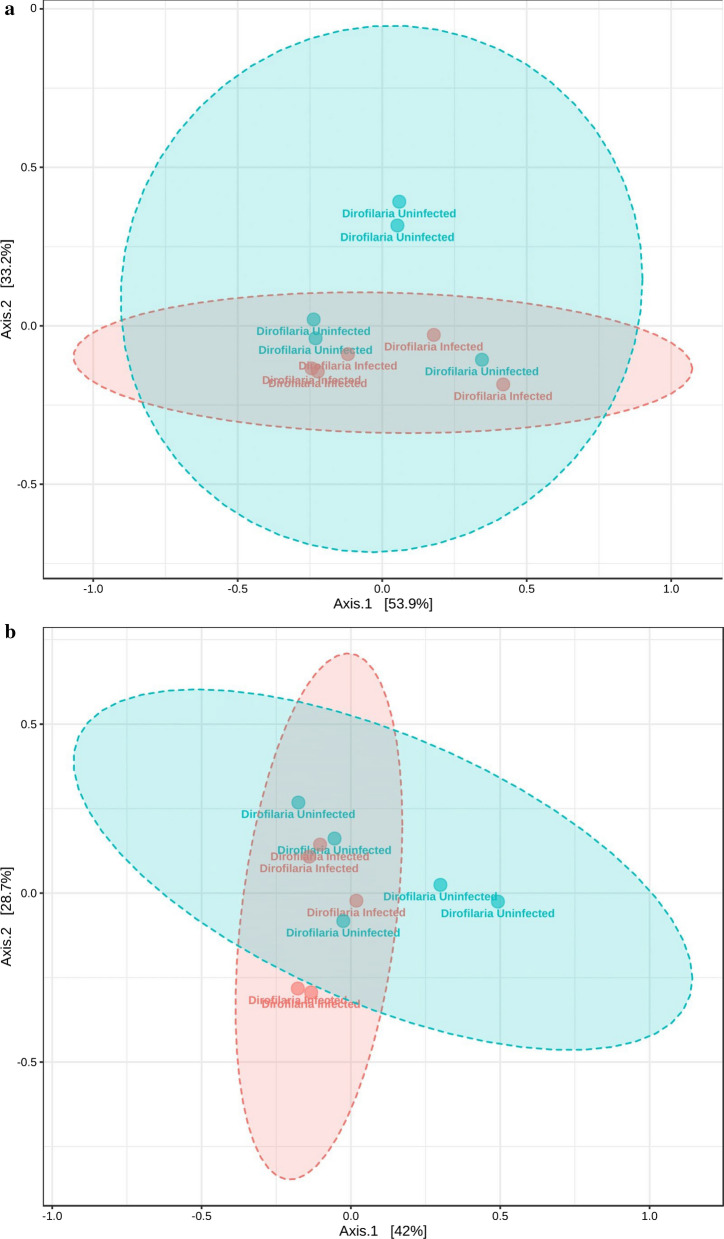
Beta diversity analysis. Estimation of differences in the microbial communities between infected and uninfected mosquitoes using principal coordinates analysis (PCoA) of the unweighted UniFrac (**a**) (PERMANOVA, pseudo-*FDR* = 1.4043, *df* = 1, *R* = 0.14932, *P* = 0.286) and Bray-Curtis (**b**) (*P* < 0.05) distance matrices

We also visualized the similarities and differences in the microbial composition of infected and uninfected mosquitoes by carrying out principal coordinates analysis (PCoA) of the unweighted UniFrac and Bray-Curtis distance matrices (Fig. 5c, d). Figure 5c, d shows distinct clustering of *D. immitis-*infected mosquito replicates with no outliers. Beta diversity was significantly changed in *D. immitis-*infected mosquitoes compared to uninfected mosquitoes using the Unweighted UniFrac distance matrix (PERMANOVA, pseudo-*FDR* 1.4043, *R*^2^ = 0.14932, *P* = 0.286).

### Community profiling and correlation analysis

**Fig. 6:**
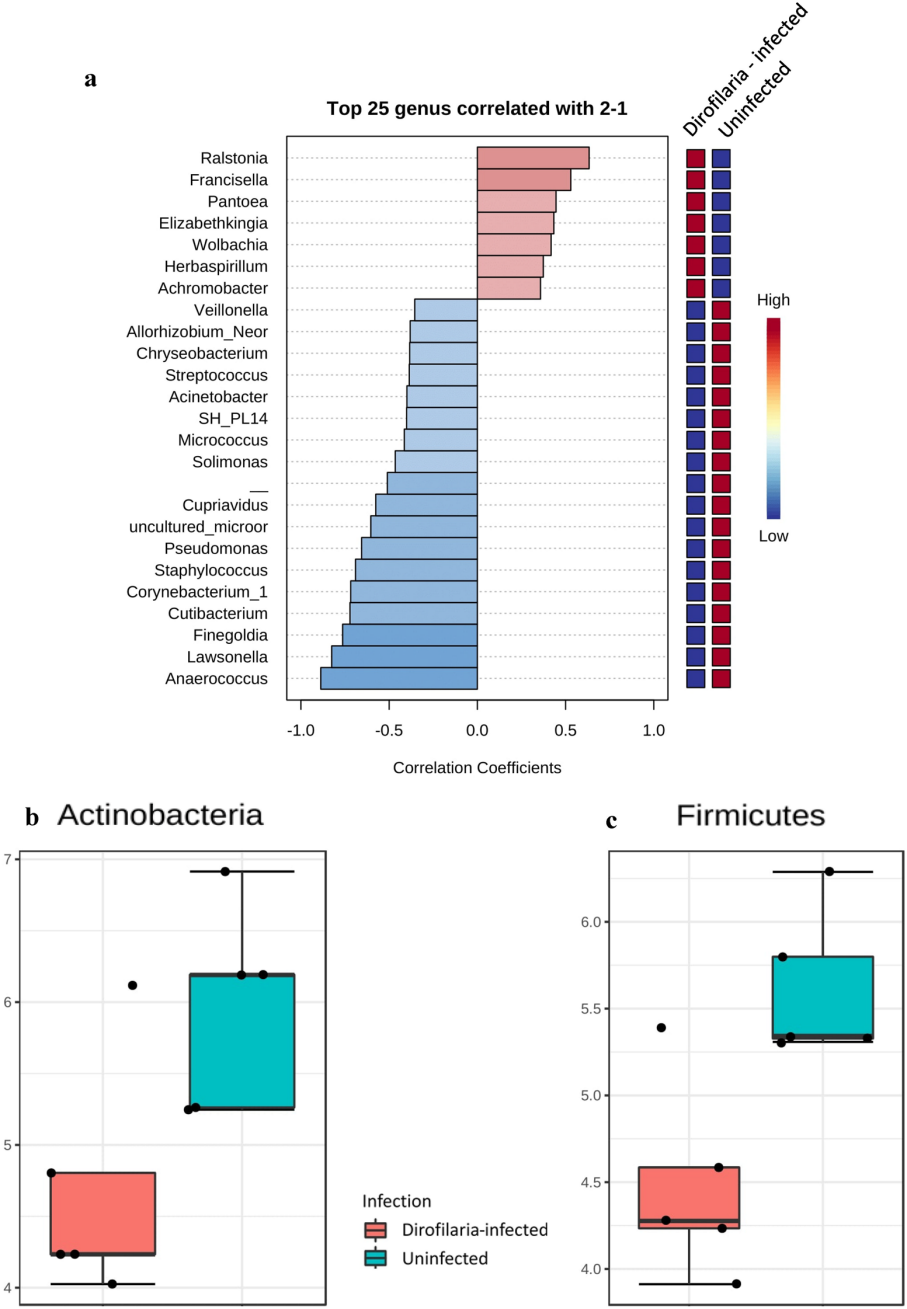
Microbial community profile. Pattern correlation analysis and significant abundance analysis of top taxa identified in *Dirofilaria immitis-*infected and uninfected mosquitoes. SparCC correlation of top 25 genera showing bacteria with strong positive correlation with *Dirofilaria immitis-*infected mosquitoes (**a**), log-transformed count of bacteria of the phyla Actinobacteria (**b**) and Firmicutes (**c**) with significant differences in abundance between infected and uninfected mosquitoes (**b** FDR = 0.1440, *df* = 1, *P* = 0.036; **c** FDR = 0.14401, *df* = 1, *P* = 0.005)

To assess the extent to which highly abundant bacteria phyla and genera were represented in *Ae. aegypti*, we used a combination of pattern correlation and heat map analysis. A very strong positive correlation was seen between the genera *Ralstonia*, *Francisella*, *Pantoea*, *Elizabethkingia*, *Wolbachia*, *Herbaspirillum* and *Achromobacter* and *D. immitis-*infected mosquitoes (Fig. 6a). Heat map analysis and phylogenetic tree of the highly represented and dominant bacterial genera also showed the above identified genera to be well represented in more than one of the *D. immitis-*infected mosquitoes (Additional file 1: Figures S8 and S9).

**Fig. 7:**
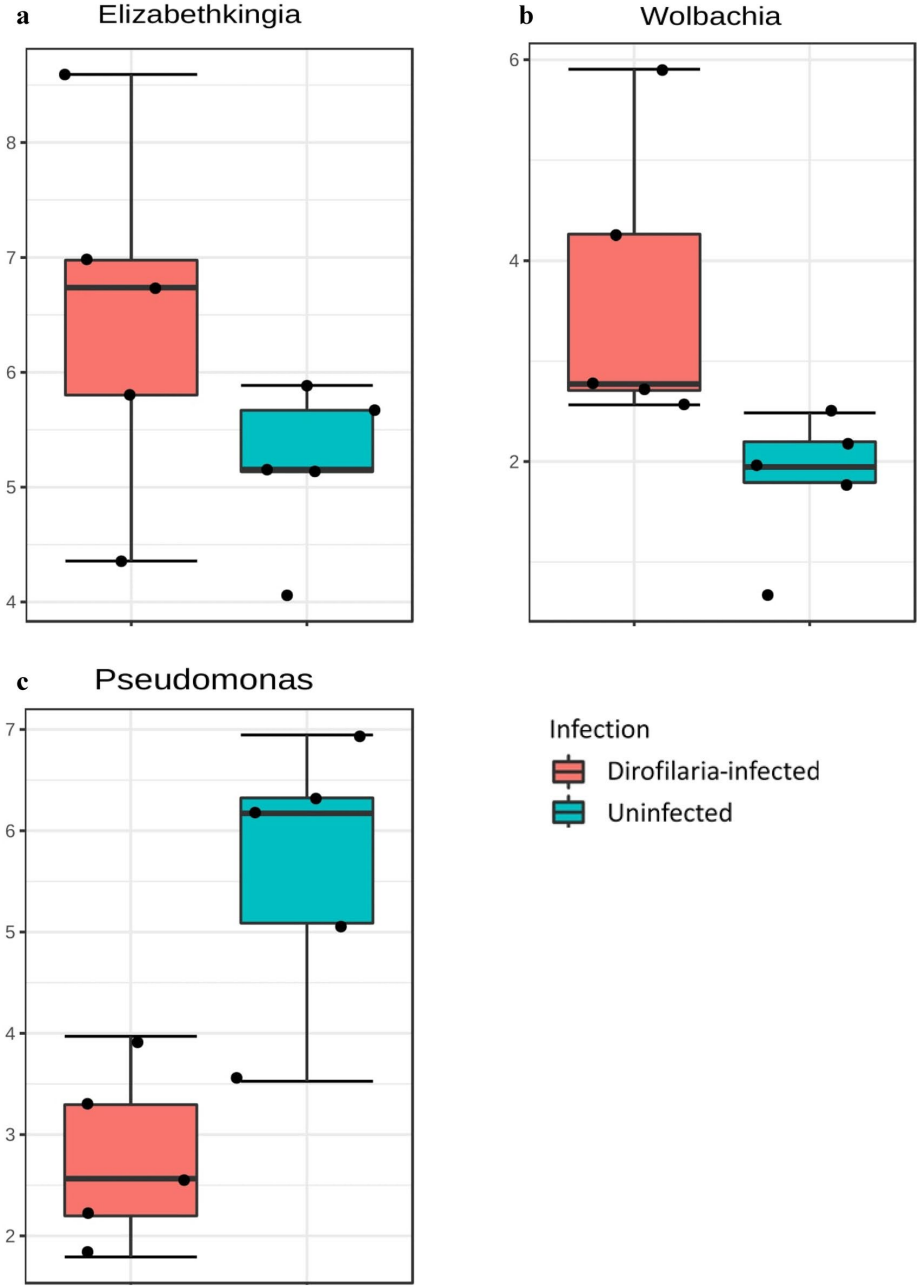
Univariate analysis. Comparison for bacteria genera displaying significant differences in relative abundance between *Dirofilaria immitis-*infected and uninfected mosquitoes. *Elizabethkingia* (FDR = 0.175, *df* = 1, *P* = 0.015) and *Wolbachia* (FDR = 0.11448, *df* = 1, *P* = 0.011) were significantly more abundant in infected compared to uninfected mosquitoes (**a**, **b**), while uninfected mosquitoes had more *Pseudomonas* (FDR = 0.12698, *df* = 1, *P* = 0.015) (**c**) compared to infected mosquitoes

To explore how top taxa differed between both infected and uninfected mosquitoes, classical univariate statistical comparisons analysis was applied to identify phyla and genera that exhibit significant differences (Mann–Whitney test) in their composition. The phyla Actinobacteria (FDR = 0.1440, *P* = 0.036) and Firmicutes (FDR = 0.14401, *P* = 0.005) had significantly higher abundance in the uninfected mosquitoes (Fig. 6b, c). Our analysis also showed that the *D. immitis-*infected mosquitoes had significantly higher abundance of the genera *Elizabethkingia* (FDR = 0.175, *P* = 0.015) and *Wolbachia* (FDR = 0.11448, *P* = 0.011), while *Pseudomonas* (FDR = 0.12698, *P* = 0.015) was much abundant in the uninfected mosquito (Fig. 7a–c).

## Discussion

To the best of our knowledge, this study is the first to utilize *16S* metagenomic analysis to understand the significance of a filarial nematode on the microbiome of a mosquito vector under laboratory conditions. This technique offers the advantage of detecting both culturable and unculturable pathogenic and non-pathogenic microbes from a given DNA or RNA sample. As previously reported from previous metagenome studies on mosquitoes and similar arthropods or insects [37–39], Fig. 1a shows that the phyla Proteobacteria, Bacteroidetes, Actinobacteria and Firmicutes were detected from the mosquito groups tested. Similar studies have also identified the phylum Proteobacteria as one of the dominant phyla in the microbiome of *Ae. aegypti* mosquitoes [23, 40]. We observed an inverse relationship in the abundance of the phyla Proteobacteria and Bacteroidetes in our study with *D. immitis-*infected mosquitoes having a higher abundance of the phylum Proteobacteria when compared to the uninfected mosquitoes and *vice versa* (Additional file 1: Figure S4).

The Proteobacteria group are the largest phylum found in different environments, plants and animals [41] with members ranging from free-living commensals to pathogenic microbes. We found that the genus *Klebsiella* was present in relatively similar abundances in both infected and uninfected mosquitoes. Although no known function has been associated to this group in insects or arthropods, *Klebsiella* belongs to a class of gram-negative Proteobacteria (Class Gammaproteobacteria) with previous detection in field- and laboratory-raised *Culex quinquefasciatus*, *Ae. albopictus* and *Ae. aegypti* [42–44].

We also identified the genus *Enterobacter* in *D. immitis-*infected mosquitoes at a relative abundance of 20-fold compared with the uninfected mosquitoes (Fig. 2). This genus has been detected in similar microbiome studies of *Ae. aegypti* where they have been associated with their role in blood-meal digestion due to their hemolytic activities [45] which could explain why the genus *Enterobacter* has been commonly associated with females of other mosquito species [46]. The role(s) *D. immitis* colonization and infection density plays in increasing the abundance of *Enterobacter* was beyond the scope of this study [47]; an elegant study by Cirimotich et al. [48] showed the inhibition of *Plasmodium* infection in *Anopheles gambiae* mosquitoes mediated by a bacterium designated as *Enterobacter* Esp_Z. The inhibition was due to the production of reactive oxygen species (ROS) by the bacteria. A pro-pathogen role was also recently associated with the genus *Enterobacter*, as it was shown that they produce Enhancins or Enhancins-like proteins, which facilitate pathogen colonization by degrading the peritrophic matrix [44]. If bacteria such as *Enterobacter* can also block *D. immitis* colonization by inducing ROS production will be interesting to see. *Enterobacter hormaechei* is another bacterium that demonstrated increased abundance in *D. immitis* infection as shown in Fig. 3. The genus *Enterobacter* have been proposed to aid in blood digestion in hematophagous insects due to their hemolytic activities [47]. Several reports have also identified different mosquito refractoriness or susceptibility to pathogen infection in the presence of different *Enterobacter* species. Cirimotich et al. [48] reported refractoriness of *An. gambiae* to *Plasmodium* infection in the presence of an *Enterobacter* species. Another species of *Enterobacter* (*E. cloacae*) was also reported to express a mucin-degrading Enhancin protein that breaks down the mucin component of the *Ae. aegypti* peritrophic matrix [44] although this was not shown to facilitate dengue virus infection.

Another bacteria genus that also increased with the presence of *D. immitis* infection is *Elizabethkingia*. Previous reports have identified this genus from the midgut of laboratory-reared [42] and field-collected [40] *Ae. aegypti*. These studies did not associate any known role to this bacterium. Bacteria in the genus *Chryseobacterium*, *Ochrobactrum*, *Sphingobacterium* and *Pseudomonas* were all present in higher abundance in the uninfected mosquito group. The detection of *Chryseobacterium* in our study agrees with similar detection in previous reports in laboratory-reared mosquitoes [49, 50]. *Pseudomonas*, a gram-negative Gammaproteobacteria was previously shown to have reduced abundance in *Wolbachia-*positive *Ae. aegypti* mosquitoes. A similar observation was also made in our study where the abundance of *Pseudomonas* was inversely correlated with the presence of *D. immitis* infection in the mosquito as shown in Fig. 2. Bahia et al. [51] reported blocking of the *Plasmodium* parasite by *Pseudomonas putida* isolated from *An. gambiae.*

Unexpectedly, only few bacteria species were differentially altered in *D. immitis-*infected and uninfected mosquitoes. *Klebsiella oxytoca*, a Gammaproteobacteria, was found at relatively similar abundance in infected and uninfected mosquitoes (Fig. 3). A study reported the detection of *K. oxytoca* from laboratory-reared and field-collected *Cx. quinquefasciatus* and *Ae. aegypti* [42]. The maintenance of this species by arthropods in both natural and artificial conditions could indicate an important role played by the bacteria. Another study reported that *K. oxytoca* reverse radiation induced loss of copulatory maintenance in male *Ceratitis capitata* [52]. A study on the fungicidal effects of bacterial colonies found on the domestic housefly eggs revealed that fungal growth is inhibited by the presence of *K. oxytoca* by producing antifungal metabolites and nutrient depletion [53]. Put together, we are proposing *K. oxytoca* as an important bacterium for *Ae. aegypti* with a likely endosymbiotic role, though further studies are still required to understand the specific roles of *K. oxytoca* in the mosquito biology including how it is maintained in the mosquito.

Another interesting observation from this study was the inverse correlation between *D. immitis* infection and microbial diversity and richness. In mosquitoes, the innate immune response is activated in the presence of invading pathogens as activation of the toll pathway and production of reactive oxygen species have been reported in mosquitoes challenged with *Plasmodium* [54] and filarial nematodes [55]. These immune effectors while countering pathogens, could in extension disrupt the normal mosquito microbiome community which could explain the overall reduction in the microbial richness observed from this study as shown in Fig. 4b, c. Our observation of reduced microbial richness in infected mosquitoes was in contrast to what was reported in a previous study which shows a more diverse microbiome in *Plasmodium-*infected *Anopheles* mosquitoes [56]. While our study did not factor the effect blood meal could have on the overall outcome of the microbial richness, few studies have reported reduction in the bacterial diversity following experimental feeding on host blood in *Ae. aegypti* [57] and *An. gambiae* [50].

## Conclusions

This study fills a knowledge gap on the interaction between a mosquito vector and a filarial pathogen of veterinary significance. We were able to show that while some bacterial species were found to be present in both mosquito groups, the relative abundances of individual species changes with the infection status, with infected mosquitoes presenting a reduced microbial richness. This indicates a likely consequence of the nematode in altering favoring or inhibiting the growth of members of the bacterial community. Ongoing study focuses on the shift in the distribution of culturable bacteria taxa in infected and uninfected mosquitoes, while also comparing the effects of *D. immitis* colonization on the microbial diversity of different tissues of the *Ae. aegypti* mosquito.

## Supplementary information

**Additional file 1: Figure S1.** Agarose gel electrophoresis of PCR amplified *D. immitis* 656-bp *cox*1 mtDNA gene confirming the infection status of *Ae. aegypti*. **Figure S2.** Quality plots of forward and reverse reads. **Figure S3.** Summary of sequence reads following demultiplexing. **Figure S4.** Relative abundance of bacteria phyla across individual mosquito samples analyzed. **Figure S5.** Relative abundance of bacteria families across individual mosquito samples analyzed. **Figure S6.** Relative abundance of bacteria genera across individual mosquito samples analyzed. **Figure S7.** Relative abundance of bacteria species across individual mosquito samples analyzed. **Figure S8.** Heat map analysis of the differential composition showing most abundant bacteria genera identified in this study. **Figure S9.** Phylogenetic reconstruction of the relative abundances of bacteria genera identified from both *D. immitis-*infected and uninfected mosquitoes.

## Data Availability

The datasets supporting the conclusion of this article are included within the article and its additional files. Raw data are available from the corresponding author upon request.
