# Supplementary material for: Laboratory colonization by Dirofilaria immitis alters the microbiome of female Aedes aegypti mosquitoes
Source: Parasit Vectors. 2020 Jul 13;13:349. doi: 10.1186/s13071-020-04218-8 (PMC7359625; doi:10.1186/s13071-020-04218-8)
Supplement: Supplementary file 1 — Additional file 1: Figure S1. Agarose gel electrophoresis of PCR amplified D. immitis 656-bp cox1 mtDNA gene confirming the infection status of Ae. aegypti. Figure S2. Quality plots of forward and reverse reads. Figure S3. Summary of sequence reads following demultiplexing. Figure S4. Relative abundance of bacteria phyla across individual mosquito samples analyzed. Figure S5. Relative abundance of bacteria families across individual mosquito samples analyzed. Figure S6. Relative abundance of bacteria genera across individual mosquito samples analyzed. Figure S7. Relative abundance of bacteria species across individual mosquito samples analyzed. Figure S8. Heat map analysis of the differential composition showing most abundant bacteria genera identified in this study. Figure S9. Phylogenetic reconstruction of the relative abundances of bacteria genera identified from both D. immitis-infected and uninfected mosquitoes. [file 13071_2020_4218_MOESM1_ESM.docx]

**Additional file 1**

**
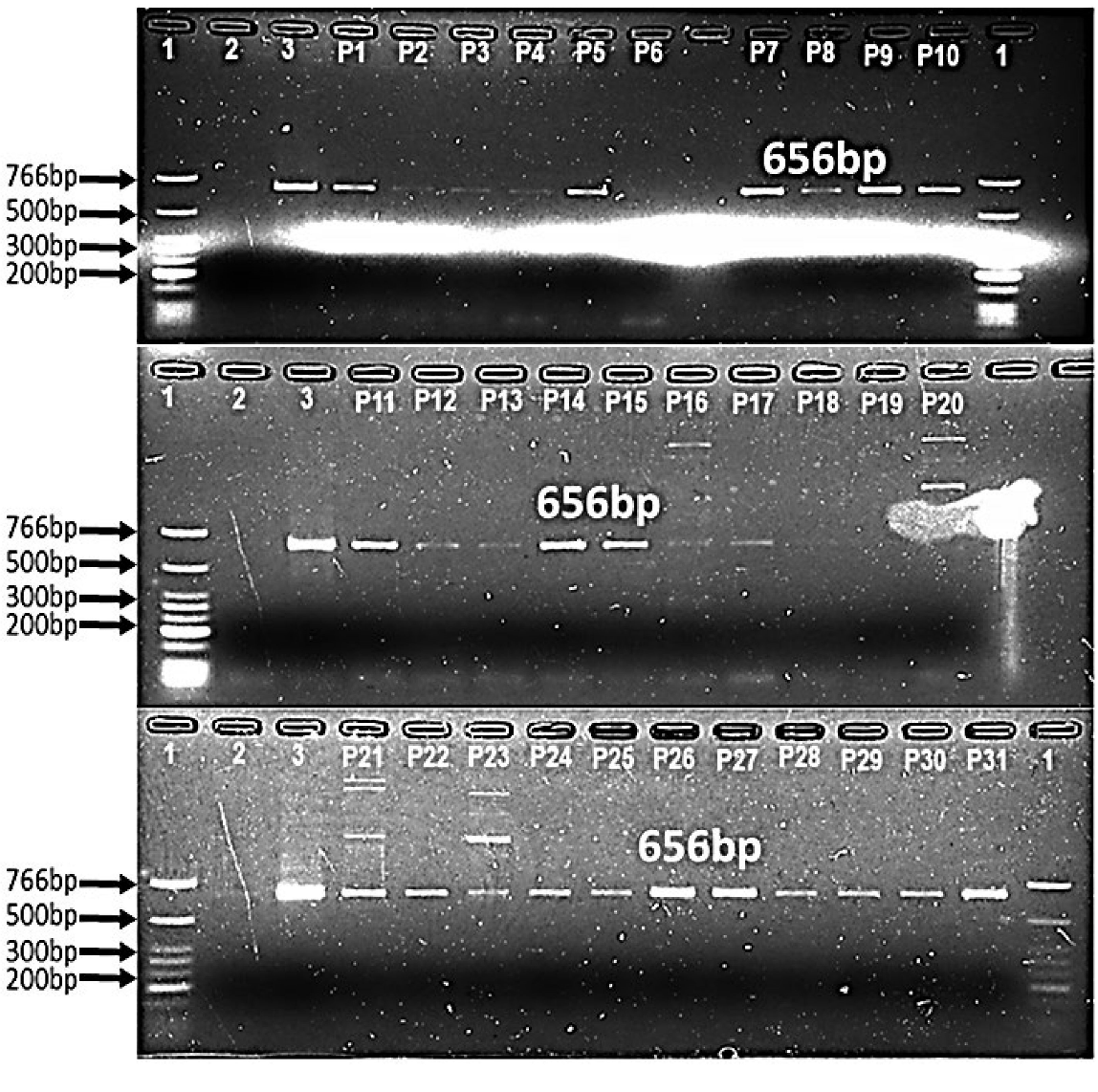
**

**Additional file 1: Figure S1. Agarose gel electrophoresis**. Gel electrophoresis of PCR amplified *D. immitis* 656bp COI mtDNA gene confirming the infection status of *A. aegypti*. Lanes 1; Low molecular weight DNA ladder, 2; negative control, 3; positive control and infected lanes represented by positive bands that corresponds to the 656bp size.

**
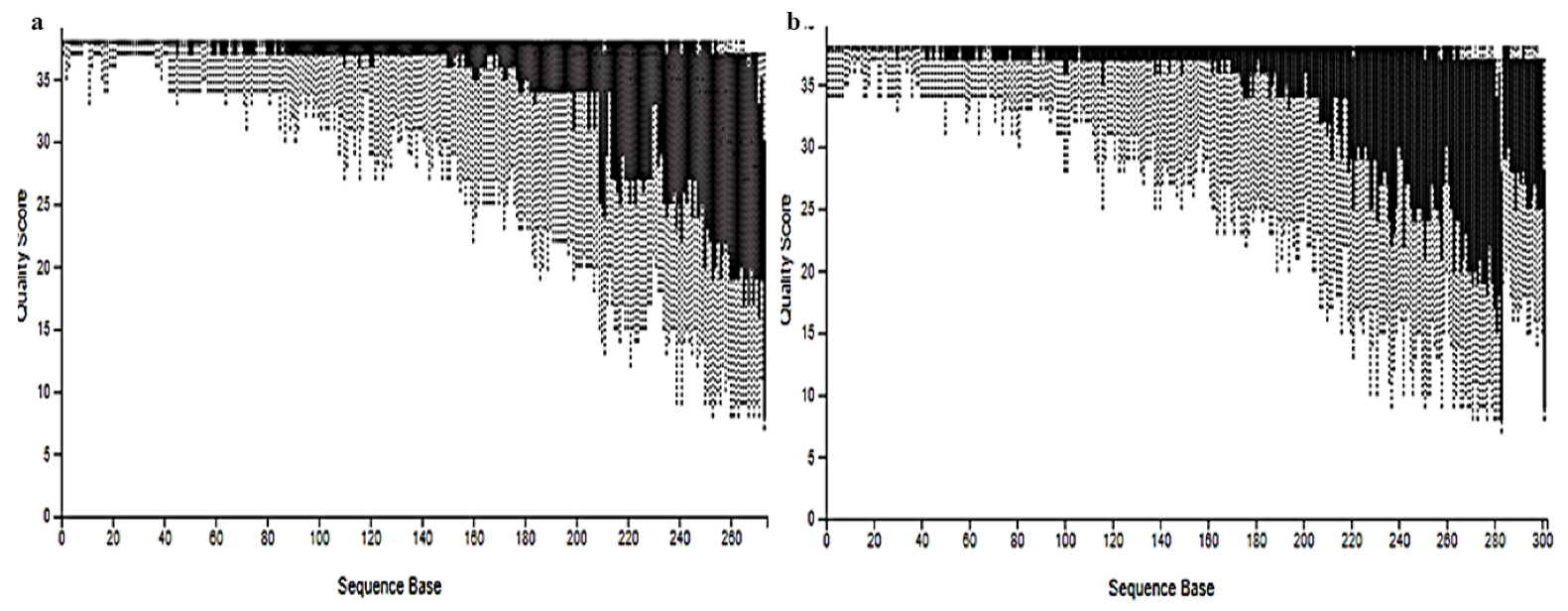
**

**Additional file 1: Figure S2. Quality plots of forward and reverse reads.** Interactive quality plots of (a), forward and (b), reverse reads. These plots were used to deduce the sequence with reduced qualities which were eventually truncated and trimmed using the DADA2 pipeline in Qiime2. The plots were generated using a random sampling of 10000 out of 602502 sequences without replacement. The minimum sequence length identified during subsampling was 59 bases for the forward reads and 87 bases for reverse reads.


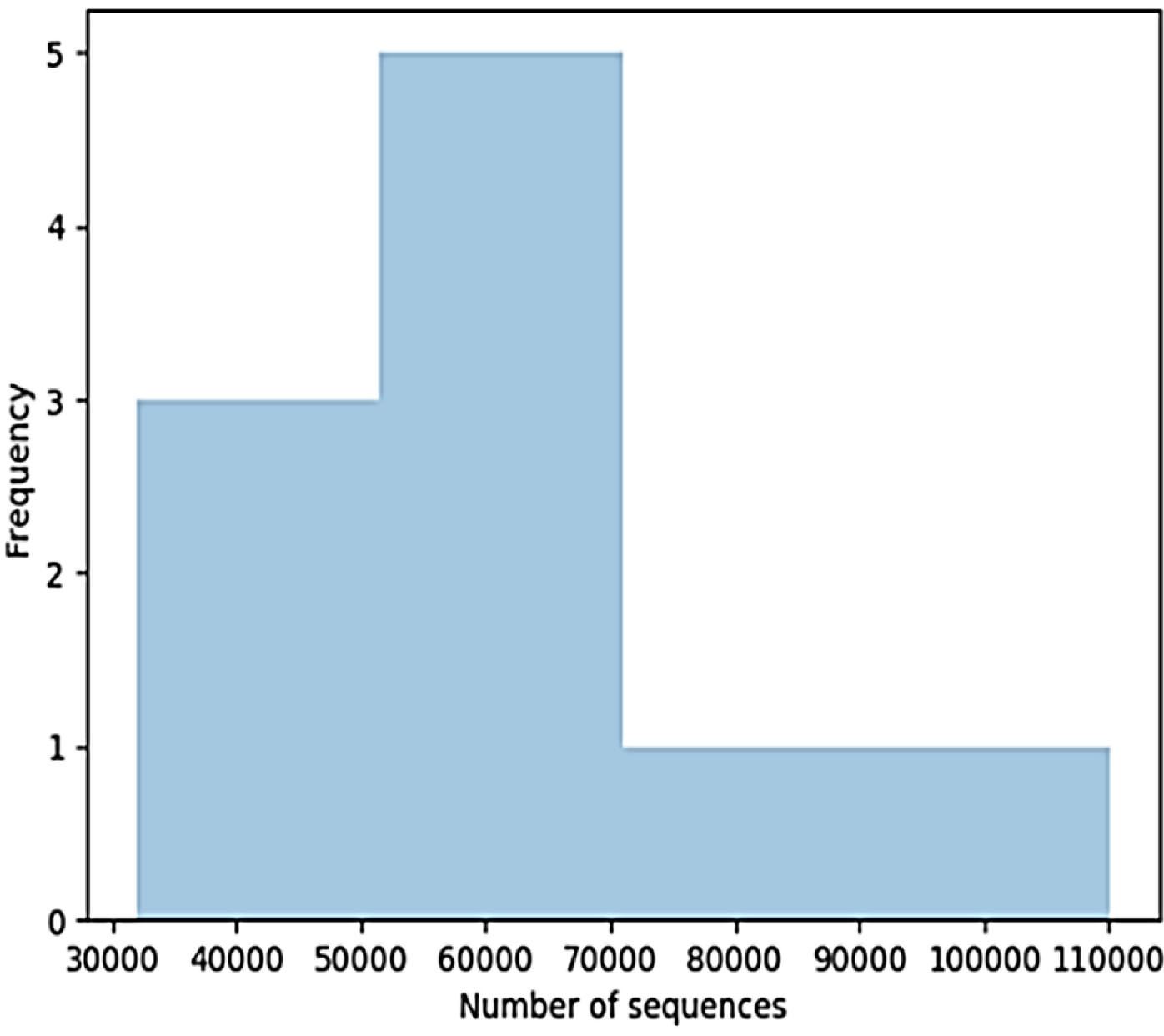


**Additional file 1: Figure S3. Summary of sequence reads following demultiplexing**. The chart represents the distribution of reads ranging from the minimum (31,861) to maximum (110,235). A total of 602502 reads were generated.


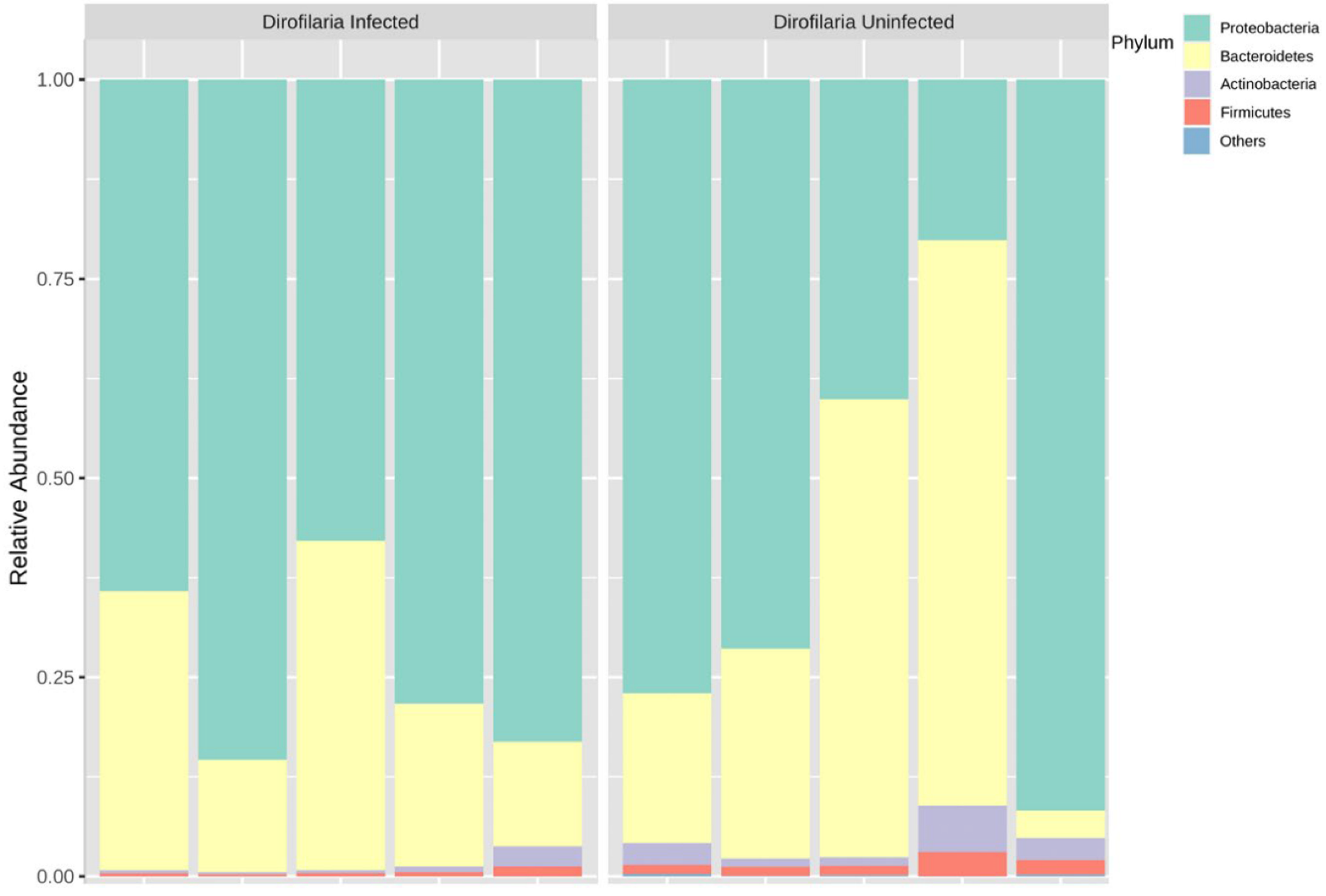


**Additional file 1: Figure S4.** Relative abundance of bacteria phylum across individual mosquito samples analyzed in this study.


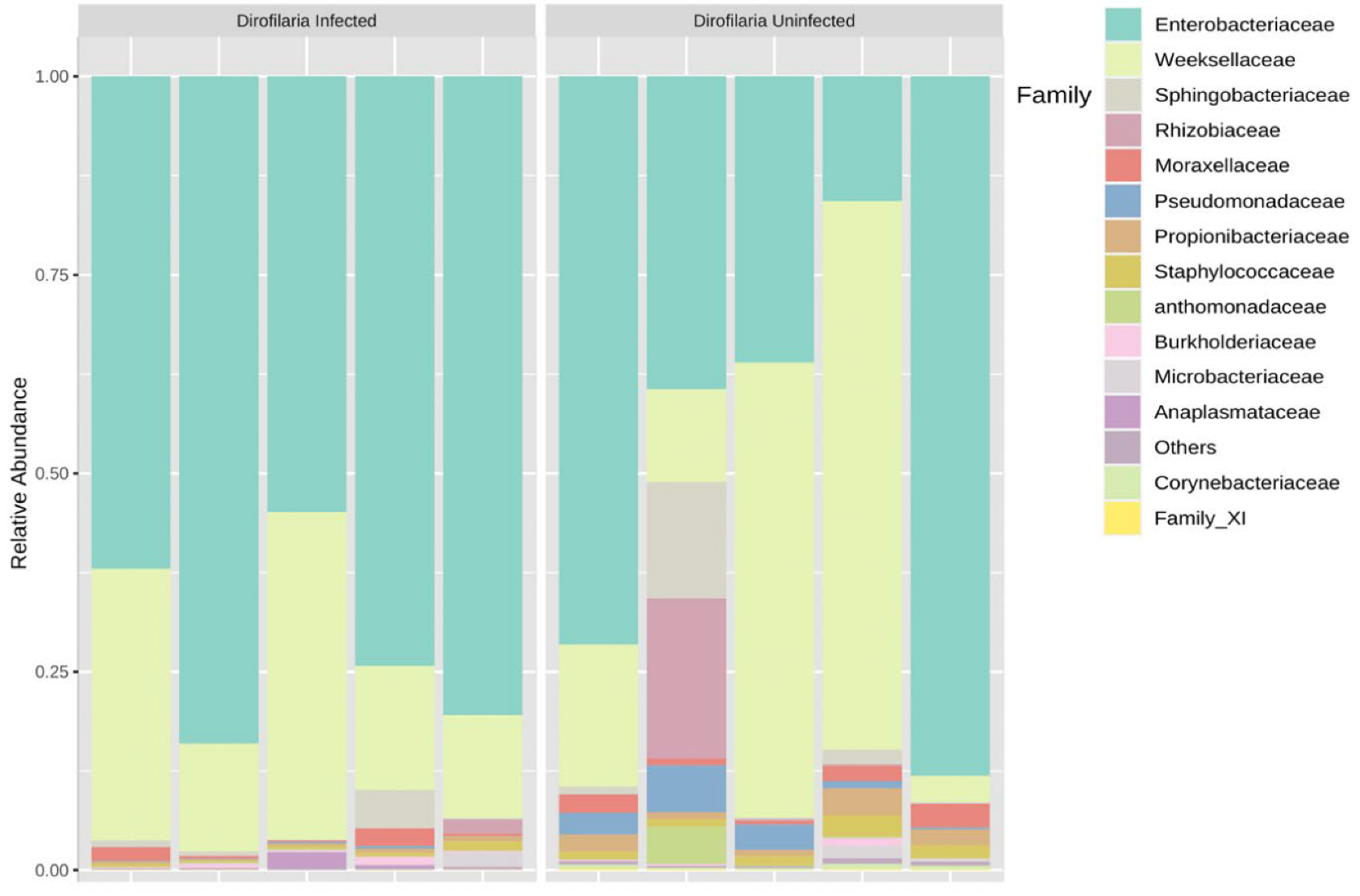


**Additional file 1: Figure S5.** Relative abundance of bacteria family across individual mosquito samples analysed in this study.


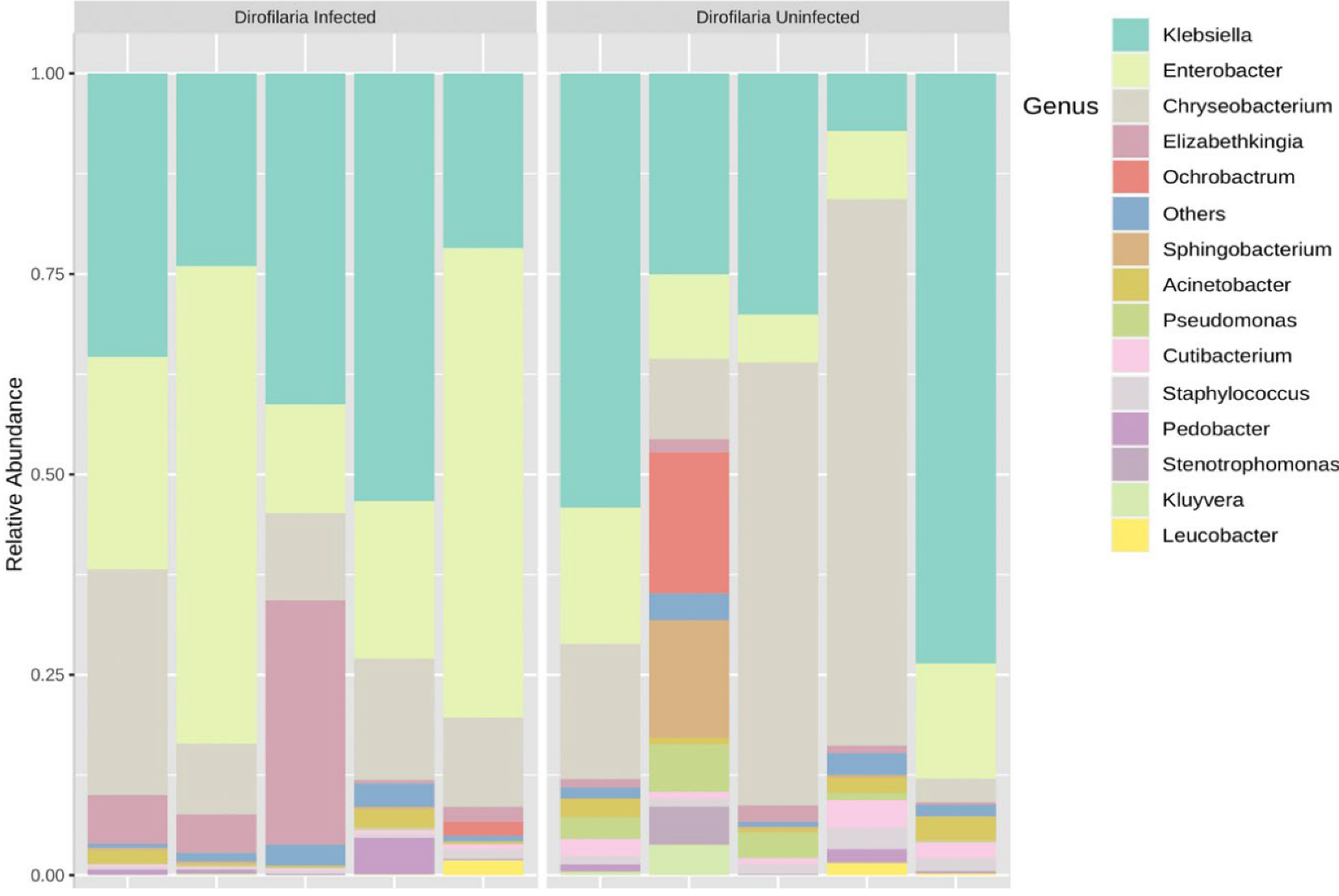


**Additional file 1: Figure S6.** Relative abundance of bacteria genus across individual mosquito samples analysed in this study


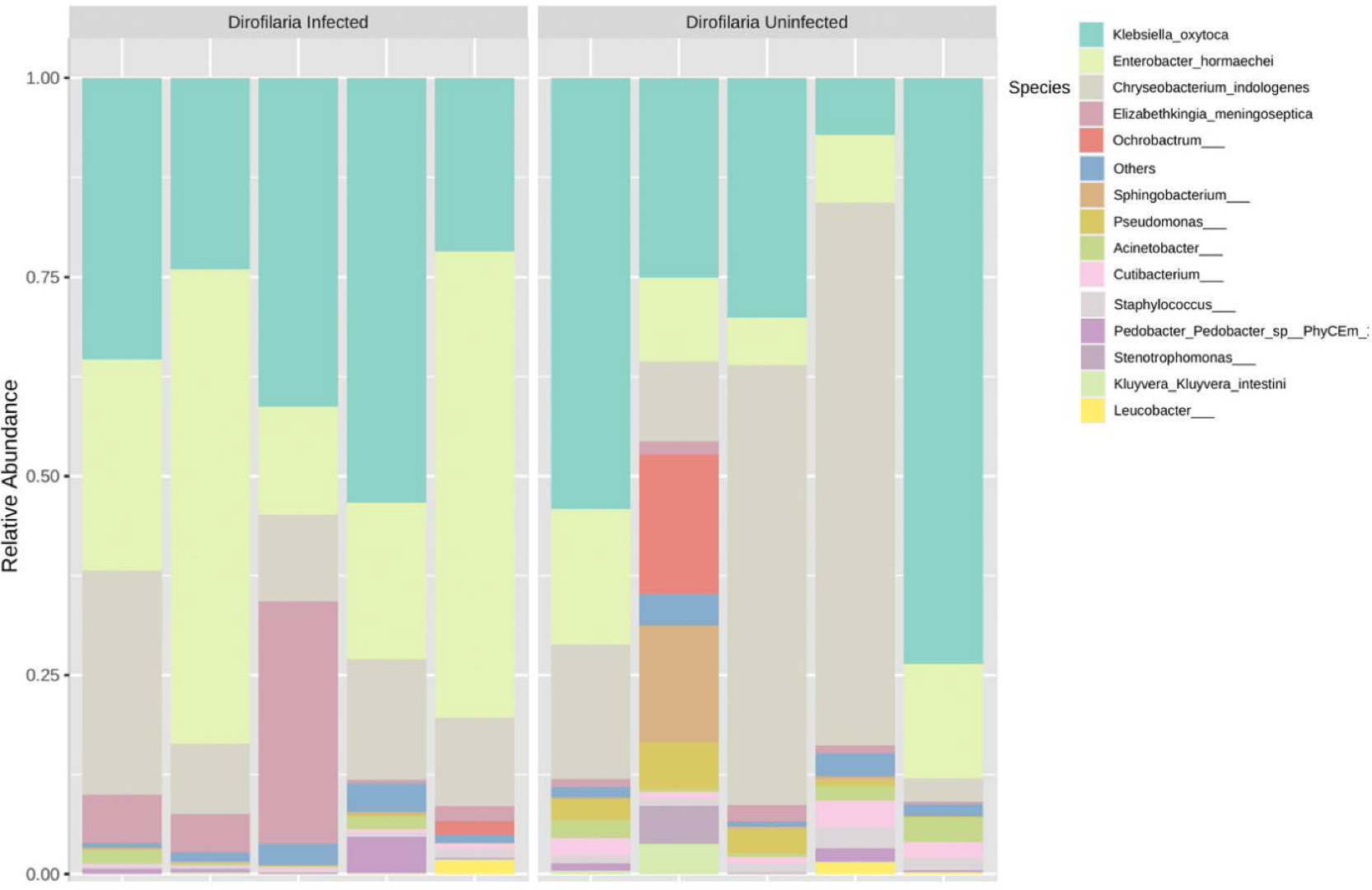


**Additional file 1: Figure S7.** Relative abundance of bacteria species across individual mosquito samples analysed in this study.


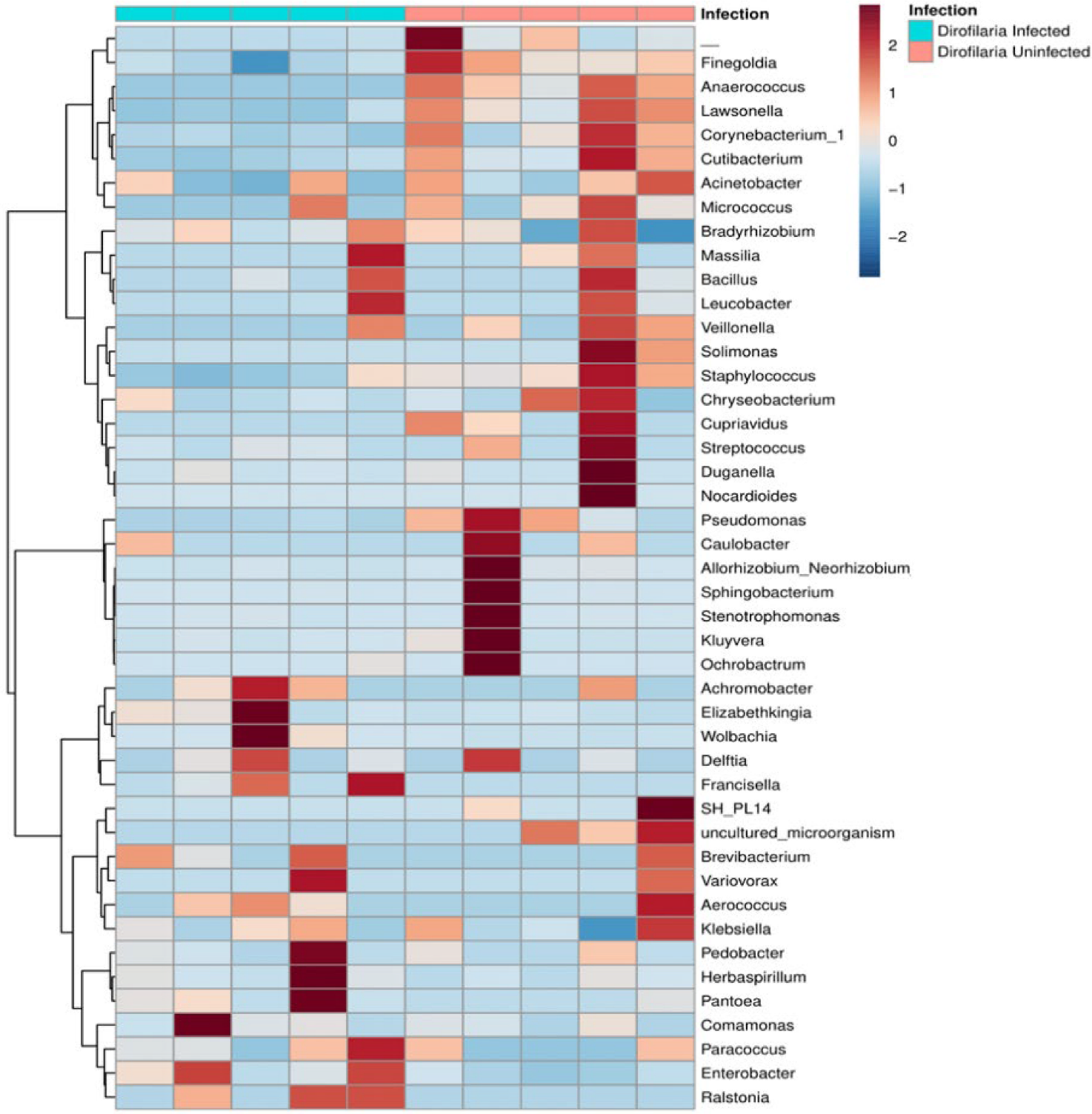


**Additional file 1: Figure S8. Heat map analysis** of the differential composition showing most abundant bacteria genus identified from this study. Mosquito samples that are indicated by a very deep red shade indicates a mosquito were identified with high abundance of the corresponding bacteria, while the deep blue color indicates low abundance.


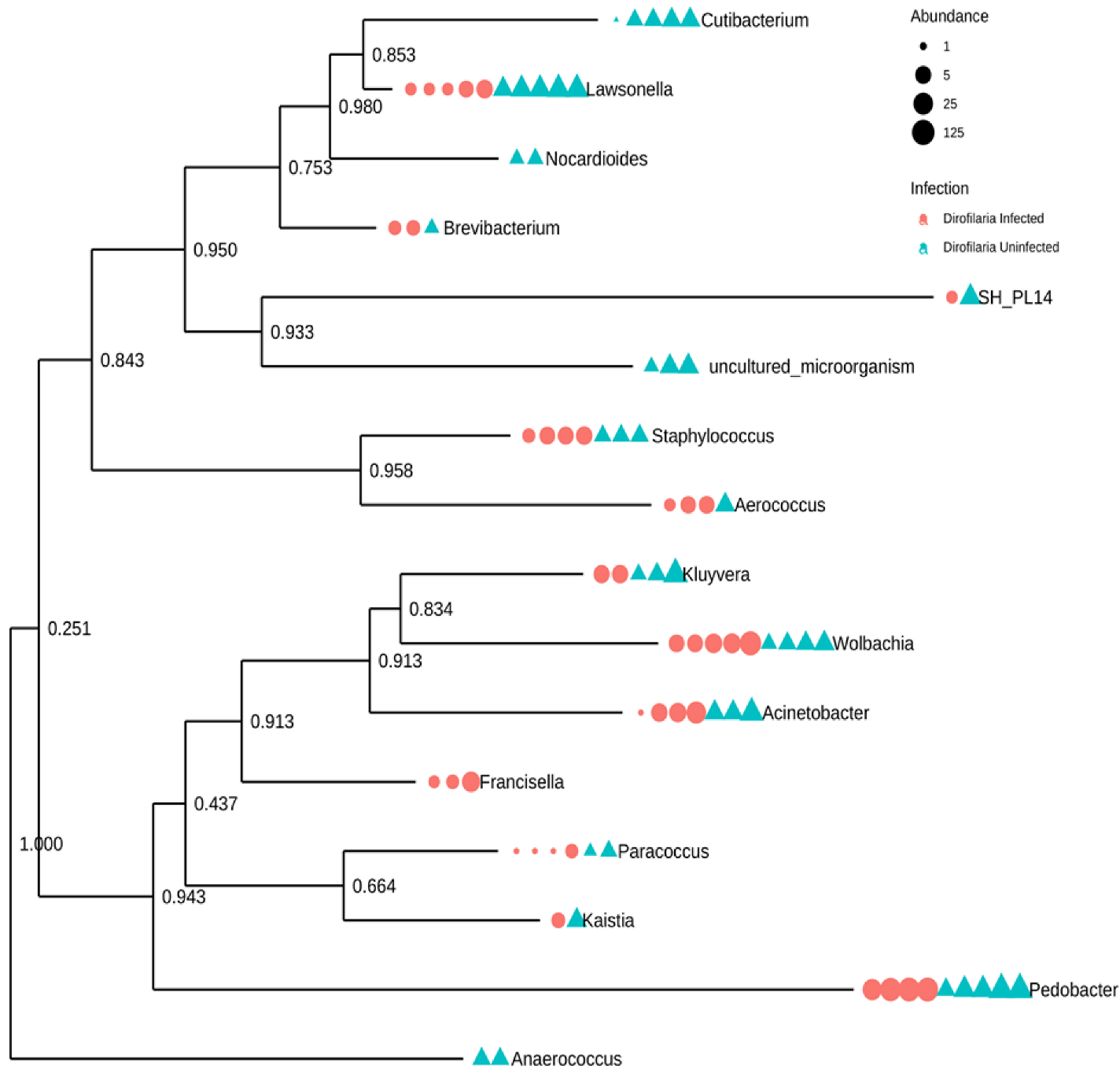


**Additional file 1: Figure S9. Phylogenetic reconstruction** of the relative abundances of bacteria genus identified from both *D. immitis* infected and uninfected mosquito.
